# Attitudes about and practices for skin cancer prevention among patients with dermatological issues in Hanoi, Vietnam: a cross-sectional study

**DOI:** 10.1186/s12199-020-00875-4

**Published:** 2020-08-01

**Authors:** Trang H. T. Nguyen, Bach X. Tran, Sau H. Nguyen, Carl A. Latkin, Cuong T. Nguyen, Son H. Nguyen, Hai Q. Pham, Cyrus S. H. Ho, Roger C. M. Ho, Jin-Kyoung Oh

**Affiliations:** 1 Department of Cancer Control and Population Health, National Cancer Center Graduate School of Cancer Science and Policy, Goyang, Republic of Korea; 2 Department of Health Economics, Institute for Preventive Medicine and Public Health, Hanoi Medical University, Hanoi, 100000 Vietnam; 3 Bloomberg School of Public Health, Johns Hopkins University, Baltimore, MD USA; 4 National Hospital of Dermatology and Venereology, Hanoi, Vietnam; 5 Department of Dermatology and Venereology, Hanoi Medical University, Hanoi, Vietnam; 6 Institute for Global Health Innovations, Duy Tan University, Da Nang, Vietnam; 7 Faculty of Medicine, Duy Tan University, Da Nang, Vietnam; 8 Center of Excellence in Health Services and System Research, Nguyen Tat Thanh University, Ho Chi Minh City, Vietnam; 9 Department of Psychological Medicine, National University Hospital, Singapore, Singapore; 10 Department of Psychological Medicine, Yong Loo Lin School of Medicine, National University of Singapore, Singapore, Singapore; 11 Institute for Health Innovation and Technology (iHealthtech), National University of Singapore, Singapore, Singapore

**Keywords:** Skin cancer, Attitudes, Practices

## Abstract

**Background:**

Raising awareness and educating people regarding practices for skin cancer or melanoma prevention are critical in the context of the adversely increasing effects of global climate change. This study aimed to explore the knowledge, attitudes, and practices regarding skin cancer prevention and to determine the associated factors to knowledge, attitudes, and practices among dermatological patients in Vietnam.

**Methods:**

This cross-sectional study included 590 dermatological patients between 18 and 82 years of age, who received an examination or treatment from the National Hospital of Dermatology in Hanoi, Vietnam, from September to December 2018. The respondents’ attitudes on skin cancer and cancer prevention were assessed via face-to-face interviews with a structured questionnaire conducted by trained interviewers.

**Results:**

Of the 590 respondents, the majority of people had correct responses to the question regarding skin cancer knowledge. Among the total participants, 39.8% thought that they were at risk of skin cancer, and 13.8% believed their occupation increased their skin cancer risk. The majority of respondents used hats (94.9%) and sunscreen skin coats (89.5%) and went into the shade (86.3%) when exposed to the sun. Women were less likely to be aware of their skin cancer risk but were more likely to practice prevention behaviors.

**Conclusion:**

Our results show that dermatological patients have acceptable knowledge towards skin cancer prevention, but still need to change their behavior to prevent the risk of skin cancer. This study highlights the importance of education to raise awareness regarding skin cancer in order to promote practice prevention strategies for skin cancer in Vietnam.

## Background

We are currently having to manage the effects of global climate change on our health, especially skin cancer, or melanoma, in which the impact is more complicated and unpredictable. Based on the report from the Global Cancer Statistic 2018, there were 300,000 new cases of melanoma globally in 2018 and it was the 15th most common cancer in men and women [1].

While the prevalence of melanoma is more common among Caucasians living in the USA and Australia, it is uncommon in Asian countries, including Vietnam [1]. Previous studies showed that the differences in the incidence rates of melanoma of the skin depended on population groups or race/ethnicity [2, 3]. Among Asians and Africans, people who have darker skin have a lower risk of skin cancer due to the presence of melanin. Melanin can be understood as a barrier to the effects of both ultraviolet (UV) A and B [4]. Vietnam is located in Asia; it is reasonable for skin cancer to be uncommon in Vietnam even though it is located in a tropical area with strong UV radiation [4]. Moreover, many studies have indicated an increasing trend in the incidence of skin cancer in European countries [5, 6], while the rates tended to be stable in Asia [5]. In Vietnam, based on the estimate from the International Agency for Research on Cancer, in 2018, there were 157 new cases of melanoma, and the mortality rate associated with this type of cancer was quite low compared with that associated with the other types of cancer (0.09% of the total cancer deaths) [1]. However, the incidence of and mortality associated with malignant skin cancer in Vietnam have remained stable in recent years.

Among the various risk factors, studies have reported that 86% of melanoma cases are caused by being exposed to UV radiation from the sunlight [7, 8]. The association between sun exposure and malignant melanoma is complicated due to the transition of the global climate as well as ozone depletion, which lead to an increase in the amount of UV [9].

Skin cancer is less common in Vietnam compared to that in other countries [1, 5, 6]; however, UV exposure is an avoidable risk factor, and skin cancer is one of the few preventable forms of cancer. Moreover, global climate change has impacted countries globally; thus, Vietnam, a tropical country, has experienced increasing UV radiation, in addition to its already prolonged and high heat sun exposure. Therefore, it is essential to raise awareness about skin cancer and the methods that can be employed to prevent it as a result of the long-term sun exposure in Vietnam.

The knowledge and awareness of skin cancer were well documented in other countries. However, there is a lack of studies on skin cancer awareness and practices in Vietnam. This study aimed to examine the awareness of and prevention practices for skin cancer among people with dermatological issues in Vietnam.

## Methods

### Study setting and subjects

A cross-sectional study was conducted at the National Hospital of Dermatology in Hanoi, Vietnam, from September to December 2018. This hospital is one of the top specialty hospitals in Vietnam and provides services for a large number of patients from Hanoi and the surrounding provinces every year. We used a convenience sampling technique to recruit participants who met the following eligibility criteria: (1) 18 years old and above, (2) received an examination or treatment from the National Hospital of Dermatology, (3) agreed to participate in the study, and (4) had a condition that they were willing to communicate with the study team about. Patients with severe illness during the recruitment process were excluded.

### Measurements and instruments

We conducted a pilot study among 20 participants of different ages, genders, and occupations to examine the acceptability and reliability of the structured questionnaire. In this pilot, the participants were requested to answer a 30-min face-to-face questionnaire. Information about the respondents’ socioeconomic characteristics was collected, including age, gender, education, marital status, occupation, living area, and monthly income. This information was then used to develop the complete questionnaire. The monthly household income of the respondents was divided into four groups, from lowest to highest. Information on the respondents’ dermatological histories included their previous diagnosis and issues. Finally, we asked respondents whether they had a family history of skin cancer and where they obtained information regarding the disease.

To gauge the respondents’ knowledge regarding skin cancer, we asked participants to report their perceptions regarding the following: (1) common levels of skin cancer in Vietnam, (2) whether skin cancer is benign or malignant, (3) the possibility of skin cancer leading to death, and (4) the effects of cloudy days on the risk of skin cancer. The primary outcomes of this study were the attitudes and practices regarding skin cancer risk and prevention. Patients reported their attitudes regarding skin cancer risk; they were asked if they consider the risk of skin cancer in their daily life and their occupation. Moreover, they were asked to report their self-assessed frequency of taking measures to protect their skin when going into the sun. In Vietnam, women prefer to use a sunscreen coat, which can cover their whole or upper part of the body for cosmetic purpose. We used Likert scales including five options from “Never” to “Always” to assess the respondents’ practices; higher scores indicated more preferable outcomes.

### Statistical analysis

We analyzed the data using STATA version 12 (Stata Corp. LP, College Station, USA). Chi-squared and Mann-Whitney tests were used for the descriptive analyses of clinical and demographic characteristics among respondents as well as the sources of skin cancer’s information. To identify the factors associated with attitudes about and practices for skin cancer prevention, multivariate logistic and Tobit regression analyses were used. We applied a forward stepwise selection strategy to eliminate non-significant factors; the significance threshold for the inclusion of variables in the multivariate analysis was a *p* value less than 0.2 based on the log-likelihood ratio test. *p* values < 0.05 were considered statistically significant.

## Results

Overall, 590 respondents were enrolled in the study; Table 1 describes the demographic characteristics of those enrolled. The mean age of the respondents was 35.5 years (SD = 12.3). The majority of participants were males (53.1%) and living in rural areas (52.3%). Most of the respondents were white-collar workers (40.1%), followed by freelancers (28.7%).

**Table 1:** Characteristics of the participants who visited a hospital with dermatological issues in Hanoi, Vietnam, 2018

Characteristics	Thinking he/she has a risk of skin cancer						***p*** value
	No		Yes		Total		
	***n***	%	***n***	%	***n***	%	
**Gender**							
Male	144	41.6	164	70.1	308	53.1	< 0.01
Female	202	58.4	70	29.9	272	46.9	
**Education**							
Under high school	54	15.3	11	4.7	65	11.1	< 0.01
High school	75	21.2	23	9.8	98	16.7	
Vocational colleges	59	16.7	23	9.8	82	13.9	
University/postgraduate	166	46.9	177	75.6	343	58.3	
**Marital status**							
Single/divorce/separation	127	35.7	59	25.2	186	31.5	0.01
Living with a partner	229	64.3	175	74.8	404	68.5	
**Employment**^**a**^							
Freelancer	134	37.7	35	15	169	28.7	< 0.01
White collar	88	24.8	148	63.2	236	40.1	
Blue collar	50	14.1	18	7.7	68	11.5	
Student	43	12.1	16	6.8	59	10.0	
Others	40	11.3	17	7.3	57	9.7	
**Living area**							
Urban	216	60.8	92	39.3	308	52.3	< 0.01
Rural	139	39.2	142	60.7	281	47.7	
**Have family members with cancer**							
Yes	11	3.1	10	4.3	21	3.6	0.46
No	339	96.9	222	95.7	561	96.4	
**Family income (Vietnamese thousands dong)**^**b**^							
Lowest (0–10.000)	140	41.08	54	24.43	194	35.53	< 0.01
Low (11.000–15.000)	52	16.0	118	53.39	170	31.14	
High (16.000–20.000)	62	19.08	26	11.76	88	16.12	
Highest (23.000–40.000)	71	21.85	23	10.41	94	17.22	
	**Mean**	**SD**	**Mean**	**SD**	**Mean**	**SD**	***p*value**
**Age**	35.9	14	34.8	9.1	35.5	12.3	0.19

^a^Employment: freelancer, people who are self-employed; white collar, office workers; blue collar, manual workers

^b^Family income: we calculated the mean monthly household income of each group

Table 2 shows the clinical characteristics of the respondents. The most common diagnosis was atopic dermatitis (42.4%), while the least common was autoimmune skin diseases (1.2%). Only 6.0% of respondents had no dermatological diseases when they participated in the interview. Overall, 30.6% of respondents had more than one dermatological issue.

**Table 2:** Clinical characteristics of the study participants who visited a hospital with dermatological issues in Hanoi, Vietnam, 2018

Characteristics	Thinking he/she has a risk of skin cancer						***p*** value
	No		Yes		Total		
	***n***	%	***n***	%	***n***	%	
**Dermatology diagnosis**							
Atopic dermatitis	99	28.3	148	63.8	247	42.4	< 0.01
Contact dermatitis	77	22	29	12.5	106	18.2	< 0.01
Psoriasis	26	7.4	4	1.7	30	5.2	< 0.01
Skin infection	20	5.7	1	0.4	21	3.6	< 0.01
Tinea	63	18	21	9.1	84	14.4	< 0.01
Autoimmune skin disease	3	0.9	4	1.7	7	1.2	0.45
Urticaria	49	14	31	13.4	80	13.7	0.83
Warts	18	5.1	10	4.3	28	4.8	0.65
Zona	32	9.1	13	5.6	45	7.7	0.12
Herpes simplex	8	2.3	5	2.2	13	2.2	0.92
STDs	8	2.3	5	2.2	13	2.2	0.92
Others	81	23.1	36	15.5	117	20.1	0.03
**Currently, have skin disease**							
No	29	8.3	6	2.6	35	6.0	0.01
Yes	321	91.7	226	97.4	547	94.0	
**Have more than one dermatological disease**							
Healthy	29	8.3	6	2.6	35	6.0	< 0.01
One disease	203	58	166	71.6	369	63.4	
More than one disease	118	33.7	60	25.9	178	30.6	

The knowledge, attitudes, and practices regarding skin cancer prevention by gender are demonstrated in Table 3. There was a similarity in the level of knowledge between males and females when answering the following true or false questions: “The risk of skin cancer decreases on cloudy days” (92.2% of males and 88.6% of females responded correctly). “Skin cancer is not common in Vietnam” (72.5% males and 65.1% females responded correctly). Regarding the attitude towards skin cancer, males were more concerned about their risk of skin cancer than females (53.2% and 25.7%, respectively). Besides, hat, umbrella, sunscreen coat, and going into the shade are the most common ways to prevent skin cancer in both sexes. However, female participants preferred to use sunscreen (75.7%), sunglasses (68.8%), and sunscreen trousers (91.9%) when compared to males (13.0%, 33.8%, and 43.5%, respectively).

**Table 3:** Knowledge, attitudes, and practices for skin cancer prevention among participants in Hanoi, Vietnam, 2018

Item	Male		Female	
	***n*** = 308	%	***n*** = 272	%
**Knowledge on skin cancer**				
Skin cancer is not common in Vietnam	241	78.2	177	65.1
Skin cancer is benign	151	49.0	214	78.7
Skin cancer can be deadly	89	28.9	114	41.9
The risk of skin cancer decreases in cloudy days	284	92.2	241	88.6
**Attitude about skin cancer**				
Thinking he/she has a risk of skin cancer	164	53.2	70	25.7
Thinking his/her occupation increases skin cancer risk	40	13.0	41	15.1
**Prevention practice for skin cancer**				
Hat	294	95.5	265	97.4
Umbrella	227	73.7	227	83.5
Sunscreen coat	258	83.8	266	97.8
Sunscreen trousers	134	43.5	250	91.9
Sunglasses	104	33.8	187	68.8
Sunscreen	40	13.0	206	75.7
Go in the shade	262	85.1	241	88.6
Take sunscreen pills	9	2.9	17	6.3

Table 4 presents the sources of skin cancer information among respondents. The most commonly used channel was the Internet (45.1%), followed by the television (22.5%) and social networks (22.0%); 20.6% of respondents received information from health workers, and only 16.9% got information from friends or relatives.

**Table 4:** Sources of information on skin cancer among participants in Hanoi, Vietnam, 2018

Characteristics	Thinking he/she has a risk of skin cancer						***p*** value
	No		Yes		Total		
	***n***	%	***n***	%	***n***	%	
Friends/relatives	78	22.2	21	8.9	99	16.9	< 0.01
Poster/banner	48	13.7	28	11.9	76	13.0	0.53
Internet	104	29.6	160	68.1	264	45.1	< 0.01
SMS/mobile phone	53	15.1	24	10.2	77	13.1	0.09
Television	94	26.8	38	16.2	132	22.5	< 0.01
Community leader	52	14.8	29	12.3	81	13.8	0.40
Paper/book	51	14.5	29	12.3	80	13.7	0.45
Health workers	82	23.4	39	16.6	121	20.6	0.05
Social network	82	23.4	47	20.0	129	22.0	0.34
Others	43	12.2	15	6.4	58	9.9	0.02

Table 5 presents the factors associated with attitudes and practices regarding skin cancer risks and prevention. Female respondents were less likely to be aware of skin cancer risks (odds ratio [OR] = 0.37; 95% confidence interval [CI] = 0.23–0.59); however, they were more likely to practice prevention behaviors than men (Coef = 5.88; 95% CI = 5.07–6.68). Compared to those who attained less than a high school degree, those who graduated from vocational colleges and university or postgraduate school were more likely to have more attention on the risk factors of skin cancer or a better attitude. Meanwhile, blue-collar workers were more likely to perceive that their occupation increases their skin cancer risk compared to freelance workers (OR = 2.73; 95% CI = 1.21–6.16). Finally, getting information about skin cancer from sources including mobile phones, the Internet, and social networks were positively associated with attitudes and practices regarding skin cancer.

**Table 5:** Factors associated with attitudes and practices for skin cancer prevention among participants in Hanoi, Vietnam, 2018

Characteristics	Thinking he/she has a risk of skin cancer		Thinking his/her occupation increases skin cancer risk		Prevention for skin cancer	
	OR	95% CI	OR	95%CI	Coef	95% CI
**Gender** (female vs male)	0.37***	0.23; 0.59			5.88***	5.07; 6.68
**Age**					− 0.04**	− 0.08; − 0.00
**Education** (vs under high school)						
Vocational colleges	1.97*	0.91; 4.28	3.56***	1.89; 6.72		
University/postgraduate	3.01***	1.52; 5.99			1.13***	0.29; 1.97
**Marital status** (living with a partner vs single/divorce/separation)			0.49**	0.26; 0.92		
**Employment**^a^ (vs freelancer)						
White collar	3.01***	1.70; 5.34				
Blue collar	2.73**	1.21; 6.16	2.43**	1.17; 5.04		
Student			0.39	0.12; 1.28		
Others	1.86	0.85; 4.04	0.22**	0.05; 0.97		
**Family income**^b^ (vs lowest)						
Low	1.85**	1.03; 3.30				
Highest					− 1.24**	− 2.30; − 0.18
**Area** (rural vs urban)	1.59*	0.93; 2.74	0.64	0.35; 1.20	− 1.29***	− 2.17; − 0.40
**Have a family member with skin cancer** (yes vs no)	3.57*	0.83; 15.36				
**Source of skin cancer information** (yes vs no)						
Friends/relatives	0.40***	0.21; 0.75				
Poster/banner			1.78	0.83; 3.83		
Internet	4.29***	2.57; 7.16				
SMS/mobile phone					1.49**	0.20; 2.78
Newspaper/magazine	1.88*	0.98; 3.62	1.73	0.84; 3.56		
Social network					2.09***	1.07; 3.10
**Skin cancer knowledge**	2.16	0.78; 5.98	2.30	0.71; 7.53		

***p < 0.01, **p < 0.05, *p < 0.1

^a^Employment: freelancer, people who are self-employed; white collar, office workers; blue collar, manual workers

^b^Family income: we calculated the mean monthly household income of each group

## Discussion

This current study found that participants who received examination or treatment from the National Hospital of Dermatology were concerned about their risk of melanoma. Respondents with higher education levels had more knowledge regarding skin cancer, and the respondents who were blue-collar workers were more concerned about their occupation increasing their risk of skin cancer.

Our study reveals that the majority of participants had atopic dermatitis and that those diagnosed with atopic eczema were more likely to think that they have an increased risk of skin cancer compared to others. These results might be because these patients had many symptoms present on the skin surface, which lead them to be more concerned about their risk of skin cancer. A previous study on atopic eczema confirmed the increased risk of squamous cell carcinoma but not malignant melanoma among atopic dermatitis patients [10, 11].

It has been well documented that those from lower income groups are more vulnerable to an increased risk of occupational skin cancer as a result of their working environment [12]. This finding is in line with a previous report of a Peruvian cohort [13]. In a study conducted among medical students, it was found that the majority of the participants knew about the symptoms of and prevention behaviors for skin cancer [14]; this is likely explained by their high levels of education. Another study in America indicated that the majority of their respondents were more concerned about tanning to make them more attractive than the risk of skin cancer [15].

The risk factors for melanoma have been well documented in various studies. Previous studies conducted in the UK considered ozone depletion and climate change as risk factors that were increasing the incidence of melanoma [16, 17]. In these studies, the authors indicated that climate change was increasing the average temperature, which forces people to change their behaviors to have more outdoor activities, thus leading to prolonged exposure to the sun [17]. Ozone depletion has caused many problems with skin cancer in the UK, Australia, America, etc. In Australia, melanoma of the skin was one of the third most commonly diagnosed cancers in men and women in 2014 [2]. Although malignant melanoma had a lower incidence rate than non-melanoma skin cancer, it was still responsible for a sixth of the mortality as a result of cancer among males in Australia [2]. In Vietnam, besides the impact of sun exposure, UV radiation, the transition of the climate, and agent orange, also known as dioxins, are also considered risk factors for skin cancer [18]. However, another study, conducted in a large cohort of Vietnam Army veterans, showed that the impact of dioxin on the non-melanoma skin cancer lesions of patients did not differ between the exposed and unexposed groups [19]. There remains a lack of evidence regarding the most common risk factors for skin cancer in Vietnam, as the majority of the previous studies were conducted in different countries, with different subjects.

Our study revealed that 42.6% of the respondents use sunscreen; 89.5% of all respondents used sunscreen coats as a method to prevent skin cancer. These findings show higher sunscreen use than that from the previously published studies; a previous study conducted in Peru found that 38.4% of the participants used sunscreen daily [13]. Surprisingly, the previous studies conducted in Australia among adolescents showed that only 64% and 26% of respondents wore sunscreen and protective clothing, respectively [20]. The differences are reasonable because the target population differed; adolescents infrequently prefer to wear extra clothing as a protection mechanism against the sun. They prefer to wear sunglasses and use sunscreen instead of wearing protective clothing [21]. Interestingly, in our study, men were more knowledgeable and concerned about the risks of skin cancer but did not practice any protective behaviors. Conversely, women were more likely to take preventive measures; however, they had fewer concerns about the risks of skin cancer. In Vietnam, women prefer to use a sunscreen coat, which can cover their whole or upper part of the body for cosmetic purpose. Compared to the finding regarding attitudes of sun exposure among outpatients in dermatology clinics in Peru, it was demonstrated that women had more knowledge about sun-protective behavior than men [13].

In this study, all respondents used at least one method to prevent sun exposure when performing outdoor activities. In Vietnam, as a result of urbanization, the motorcycle is the most convenient transportation method for residences. According to the report from the Survey Assessment of Vietnamese Youth 2009 (SAVY2), 95% of people use motorbikes as their mode of transportation in Vietnam [22]. In Vietnam, to protect themselves from strong sunlight, heat, dust, and risk of accident, most of the biker wear hat/helmet, sunscreen coat/trousers, and sunglasses: this might explain why all respondents use some protection against the sun.

We found that the Internet, social networks, and mobile phones were information sources used by respondents to gain skin cancer knowledge. Previous studies also indicated that the Internet is a vital source of information to provide knowledge about skin cancer [13, 23]. Additionally, previous studies found that the most important sources of information for skin cancer were the television and media, while another study found that the information obtained from the dermatologist and community leader was less important than that from another source [24]. This might be explained because younger people prefer searching for information on the Internet or mass media rather than in dermatological consultation.

Our study may have some implications. The lack of awareness regarding skin cancer may lead to late detections; thus, dermatologists should carefully consult patients regarding melanoma symptoms. Additionally, due to the transition of the climate, as the weather becomes hotter, people might prefer more outdoor activities than they did previously. This may increase the time of exposure to the sun, which may sequentially increase the risk of skin cancer if sun protection methods are not employed. It is necessary to implement interventions that will improve knowledge and raise awareness regarding skin cancer, especially targeting men who exhibited poor protective behaviors. Currently, there is no government-driven, organized skin cancer prevention program to raise awareness on UV protection in Vietnam. The most available source of information on UV protection is commercial advertisements for cosmetic products. As respondents were more likely to search for information about skin cancer on the Internet and mass media, organized skin cancer prevention projects at the government level should take this into account. Health promotion strategies should be developed using these platforms for the entire population.

This study was subject to some limitations that warrant mentioning. Our research used the convenience sampling technique, and the dermatology patients might have more attention towards, and knowledge on, skin cancer prevention, which may limit the generalizability of our findings. Furthermore, selection bias may have occurred because the study was conducted in the National Hospital of Dermatology in Hanoi only. The questionnaire was developed for this study but was not validated. There were some possible biases in our study, including recall bias and social desirability bias, which may pose some limitations regarding the validity of our findings.

## Conclusion

Our study found that the majority of respondents with dermatological issues had basic knowledge regarding skin cancer risks and prevention strategies. Even though men were more knowledgeable and concerned about the risks of skin cancer, they practiced fewer protective behaviors compared to women. Higher education and outdoor occupation were positively associated with increased awareness about skin cancer. Distribution of educational materials, such as brochures in hospitals, aimed at the general population and online health advice provided by health professionals are some of the measures that may be effective in raising awareness.

## Data Availability

The dataset is accessible from the corresponding author upon reasonable request.
